# Estimation of functional diversity and species traits from ecological monitoring data

**DOI:** 10.1073/pnas.2118156119

**Published:** 2022-10-18

**Authors:** Alexey Ryabov, Bernd Blasius, Helmut Hillebrand, Irina Olenina, Thilo Gross

**Affiliations:** ^a^Helmholtz Institute for Functional Marine Biodiversity, 26129 Oldenburg, Germany;; ^b^Institute for Chemistry and Biology of the Marine Environment, Univeristy of Oldenburg, 26129 Oldenburg, Germany;; ^c^Technische Universität Dresden, Institute for Forest Biometrics and Forest Systems Analysis, 01737 Tharandt, Germany;; ^d^Alfred Wegener Institute, Helmholtz Center for Marine and Polar Research, 27570 Bremerhaven, Germany;; ^e^Klaipedos Universitetas, Marine Research Institute, 92294 Klaipeda, Lithuania;; ^f^Lithuanian Environmental Protection Agency, 03201 Klaipeda, Lithuania

**Keywords:** functional diversity, ecological monitoring, data science, diffusion map, phytoplankton

## Abstract

The twin crises of climate change and biodiversity loss define a strong need for functional diversity monitoring. While the availability of high-quality ecological monitoring data is increasing, the quantification of functional diversity so far requires the identification of species traits, for which data are harder to obtain. However, the traits that are relevant for the ecological function of a species also shape its performance in the environment and hence, should be reflected indirectly in its spatiotemporal distribution. Thus, it may be possible to reconstruct these traits from a sufficiently extensive monitoring dataset. Here, we use diffusion maps, a deterministic and de facto parameter-free analysis method, to reconstruct a proxy representation of the species’ traits directly from monitoring data and use it to estimate functional diversity. We demonstrate this approach with both simulated data and real-world phytoplankton monitoring data from the Baltic Sea. We anticipate that wider application of this approach to existing data could greatly advance the analysis of changes in functional biodiversity.

## Significance

The rampant loss of biodiversity is starting to be recognized as a global crisis rivaling the climate emergency. To address this crisis, scientists need robust methods to measure the diversity in a system. Importantly, these methods should not only count species but capture the variety of different functions that the species in a system can perform. In this paper, we propose a machine learning method by which existing data from ecosystem monitoring can be reanalyzed to reveal changes of functional biodiversity over time.

Recent assessments have documented the ongoing precipitous loss of global biodiversity (1–4). More complex responses are observed on the regional scale, where stressors can lead to a transient increase in diversity (5, 6). Meanwhile, our understanding of the complex dynamical interplay of dispersal, extinctions, and speciation that has created Earth’s biological diversity and drives current dynamics is still woefully incomplete (7). Hence, the scale of the unfolding crisis and the intricacy of the dynamics involved but also, the gaps in our understanding highlight the need for large-scale biodiversity monitoring.

Even quantifying biodiversity loss still poses challenges. It has been argued that, for simplicity, global policy goals should be phrased in terms of the number of extinctions (8). Similarly to climate goals quantified by temperature increase, the number of extinctions has the benefit of being easily communicable. However, unlike climate change, where many detrimental effects are directly triggered by rising temperatures, extinction numbers are a poor indicator of biodiversity loss, where a major concern is the loss of biological functions (9).

On a fundamental level, biodiversity can be conceptualized as the genetic variation of forms, but due to the complexity of biological life, the genetic makeup is only a weak indicator of function (10, 11). Hence, for the assessment of ecosystem functioning, service provision, sustainability, and quantification of responses to stressors, robust measures of functional diversity are needed.

The need to understand functional diversity has been frequently highlighted (9, 12–15). Common measures, such as the Rao index (16, 17), compute functional diversity from pairwise functional distances between species. To compute such distances, researchers identify traits of the species under consideration and then, compute functional diversity from distances in trait space (e.g., refs. 17–21). As there is no universal definition of what constitutes a trait, it is useful to distinguish between physiological characteristics directly identified from observation (here, o-traits) and inferred traits that are inferred from data to approximate the fundamental niche axis in the system (i-traits).

Trait-based approaches provide good estimates of functional diversity but require the researcher to quantify the trait space of all organisms considered. The decision of which o-traits are relevant functional characteristics is made based on the researcher’s experience and is dependent on the group of species and functions under consideration. Some traits may be difficult to measure, and their values may be dependent on environmental conditions (22) and hence, are context dependent. In practice, these constraints mean that trait-based quantification of diversity is presently constrained to comparatively small groups of similar well-studied organisms and suffers from limited data availability.

In comparison with the manual determination of trait values, it is generally easier to quantify properties, such as species identity, biomass, and/or abundance. Long-term ecological research programs have accumulated a treasure trove of monitoring data, recording this information, with individual datasets spanning multiple decades and capturing dozens or hundreds of species. It is, therefore, attractive to infer trait values from such datasets. For example, ref. 23 used Bayesian model fitting to infer four values of o-traits from long-term time series. A natural next step is to use data analysis approaches to not only infer trait values but also, construct the i-trait axes directly from data.

Here, we propose an approach for the analysis of monitoring datasets that record the abundance or biomass of species observed in a set of samples. We use diffusion maps (24–26), a manifold learning method, to construct an i-trait space directly from these datasets. In contrast to previous work (23), this approach does not require a model or a list of known o-traits and is not limited to time series data. Instead, the diffusion map identifies both the i-trait axes and trait values solely from species biomass in samples. The functional diversity can then be computed from the pairwise distances in the i-trait space. We test this approach with a simulated dataset from a mathematical model before applying it to quantify functional diversity of phytoplankton communities in a monitoring dataset from the Baltic Sea (27). Our results show that the proposed method can reveal biologically meaningful trait information and allows for the robust and unambiguous quantification of functional diversity from monitoring data. In the dataset analyzed here, it reveals an increase in functional diversity with time that is significantly more pronounced at the coastal stations.

It is interesting to note that the data-driven i-trait approach used here is diametrically opposite to traditional ecological thinking. Many classic works observed morphological features of species, conjectured their functional relevance, and then, used this insight to predict spatial distribution. In the present paper, we go the opposite way, using spatial co-occurrence to infer functional niches and then conjecture their potential physiological basis.

## Trait Space Inference from Monitoring Data

Quantifying differences between dissimilar objects poses a fundamental challenge. Whereas we may be able to compare two songs or two paintings, it is much harder to quantify how dissimilar a certain painting is from a certain song. The same challenge is encountered in assessments of functional diversity, where it is essential to quantify how dissimilar pairs of (potentially very different) species are. To circumvent this problem, the diffusion map (24–26) builds on the idea that the dissimilarity between pairs of objects can be robustly quantified if they are sufficiently similar. By finding all such short-distance comparisons that can be made in the dataset, we obtain a set of “trusted” links between objects.

To apply this approach to functional diversity estimation, we quantify the similarities between species based solely on their abundance in monitoring samples. Our primarily notion of similarity is the Spearman correlation (28) between pairs of species across samples in the dataset, which provides an indicator of co-occurrence of species.

We follow ref. 26 and consider a comparison between two species as a trusted link if it ranks in the top 10 most similar comparisons for at least one of the two species. The trusted similarities are stored in a similarity matrix **S**, while all others are set to zero. The result is a network of trusted links that spans the entire set of species while containing only relatively short-ranged and hence, relatively accurate comparisons.

**Fig. 1.**
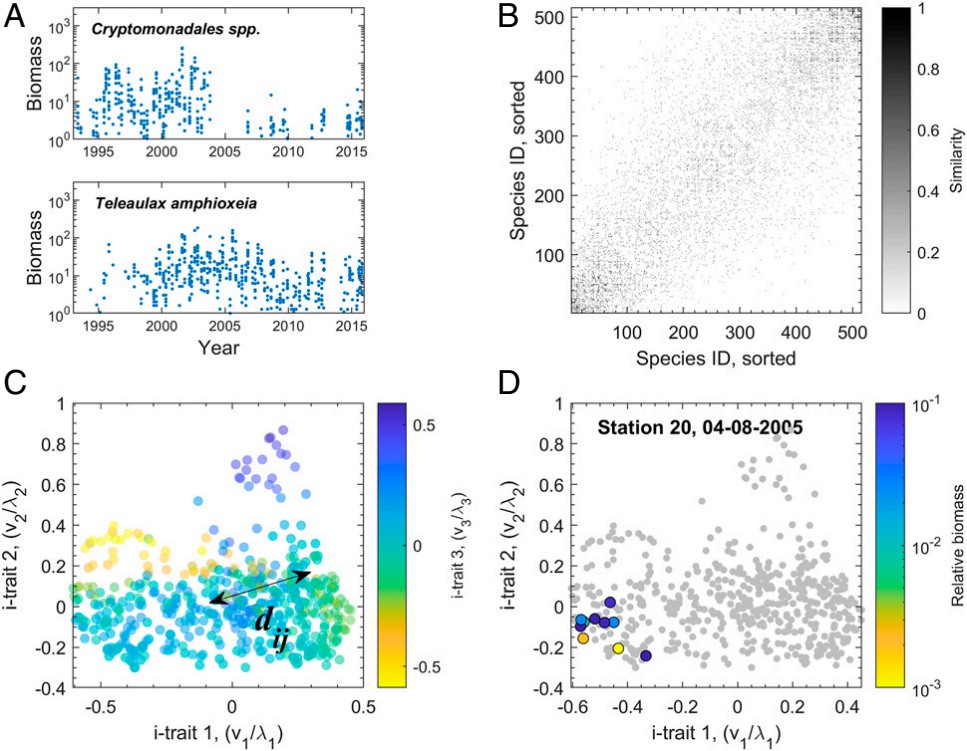
Functional diversity estimation from a monitoring dataset. (*A*) We use data on the biomass of 516 phytoplankton species in 730 samples collected from 1993 until 2015 from 10 stations in the Baltic Sea (27) (2 species are shown for illustration). (*B*) We then compute the pairwise similarity between species from the correlation between species abundances over the set of samples. (*C*) From the similarities, the i-trait space of the species (dots) is constructed using diffusion maps. In this space, the pairwise functional dissimilarity is quantified by the diffusion distance *d_ij_*. (*D*) Once the distances between species have been determined, the diversity in a specific sample can be quantified by applying the Rao’s index to the species present (highlighted dots). In the sample shown, most of the biomass is concentrated in a small area of trait space, leading to a comparatively low Rao index.

Once trusted links have been identified, we quantify the dissimilarity between species by their distance in the network of trusted links. Specifically, diffusion maps use the notion of diffusion distance (24), which takes all possible paths between network nodes into account. We use a variant of diffusion distance *d_ij_*, which can be computed efficiently from the set of eigenvectors and eigenvalues of a Laplacian matrix describing the network (*SI Appendix* has details). The result is a computationally efficient method (Fig. 1) that produces deterministic results, where our choice of trusting 10 neighbors is the only tunable parameter.

The Laplacian eigenvectors that are identified in the process are also of interest for a different reason. The *n*th eigenvector $v_{n}$ contains one element corresponding to each of the species, which is related to the *n*th i-trait for that species. The corresponding eigenvalue *λ_n_* is inversely proportional to the relative importance of this *n*th trait axis (*SI Appendix*). Hence, the rescaled vector $v_{n}/\lambda_{n}$ specifies the properly scaled value of the *n*th i-trait of all species in an effective i-trait space (Fig. 1*C*). We show below that these i-traits align well with ecological intuition.

Once the i-trait space has been constructed, we consider individual samples from the monitoring dataset (Fig. 1*D*). Building on the interspecies diffusion distances in the i-trait space, we quantify the functional diversity in the sample using Rao’s quadratic entropy (16). The method can thus quantify functional biodiversity and to some extent, place the species into a biologically meaningful trait space.

## Validation with Model Data

**Fig. 2.**
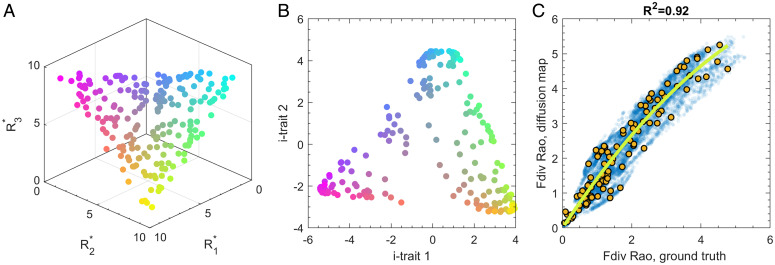
Numerical validation of the proposed method. (*A*) We numerically generate randomly distributed traits of 200 species (colored dots) that fill a triangle in a trait space spanned by three resource requirement ($R^{*}$) parameters. Color indicates the resource ratio preferred by a species. (*B*) The inferred i-trait space generated by diffusion mapping simulated biomass data. The reconstruction identifies traits that span a space that is qualitatively similar to the ground-truth traits. Colors are the same as in *A*, illustrating that neighborhood relationships are mostly reconstructed correctly. (*C*) Rao’s functional diversity calculated from diffusion distances in the reconstructed trait space correlates strongly with the numerical ground truth (based on $R^{*}$ values). Indicated are local diversity (blue dots) in individual patches and regional diversity in the metacommunity (yellow dots). The *R*^2^ value for regional diversity is 0.92, relative to a cubic regression (green line). These results show that the proposed method can be used to identify traits and robustly estimate functional diversity based on monitoring data.

To test the proposed method, we generate synthetic data by simulating a metacommunity model in silico. We consider a community of 200 primary producer species limited by three essential resources. Each species is characterized by a set of minimal resource requirements ($R^{*}$ values) reflecting the species ability to sequester the corresponding resource. We can thus envision the species as points in a three-dimensional trait space (Fig. 2*A*), where the $R^{*}$ values correspond to the traits. Specifically, we randomly draw the $R^{*}$ values such that they fill a triangular surface, modeling the existence trade-offs such that a greater ability in sequestering one resource is compensated for by a lesser ability in sequestering others.

We adapt a model from ref. 29 to simulate the population dynamics of species in 800 metacommunities, each of which consists of a square lattice of 120 discrete patches arranged in a 10×12 grid. The individual patches are characterized by random values describing the supply of three resources, mimicking real-world spatial heterogeneity, and facilitating the coexistence of model species. The biomass density of each species in each patch changes dynamically according to an equation capturing local growth and mortality as well as dispersal to and from neighboring patches (*SI Appendix*). The result is a spatial metacommunity, in which all species persisted over the duration of simulation runs.

As a first test, we consider the distribution of species in the space spanned by the two most important i-traits found by diffusion mapping the simulated biomass samples. While the i-trait space is slightly deformed in comparison with the ground truth, it retains key characteristics (compare to Fig. 2 *A* and *B*). In the i-trait space, the species still form a triangular shape on a two-dimensional surface. This result gives us confidence that the diffusion map should also be capable of inferring meaningful trait spaces from large real-world datasets.

The main purpose of the proposed method is the estimations of functional diversity. To test whether a trait space that has been inferred from one dataset can also be used to estimate the diversity in new samples, we ran 100 additional simulations and estimated the functional diversities both for the ground truth, given by the known $R^{*}$ values, and for the i-traits using the existing map. This was done both for the entire metacommunity (mimicking regional diversity) and within each patch (local diversity). A comparison of the resulting Rao indices (Fig. 2*C*) shows a strong correlation ($R^{2}=0.92$) between the ground truth and the reconstructed values of regional diversity.

We also explored how limited data availability and different distance metrics impact the accuracy of reconstruction (*SI Appendix*). The power of the diffusion map hinges on our ability to construct a spanning network of trusted comparisons between the samples. If the underlying trait space is large or the number of species is small, then we are forced to trust comparisons between comparatively dissimilar species, and the quality of the reconstruction degrades. By contrast, a larger number of observations reduces the noise in individual comparisons and improves the quality reconstruction (*SI Appendix*, Figs. S3 and S5).

In summary, results from the numerical experiments show that given a sufficient volume of data, the diffusion map can infer the trait space from a monitoring dataset. Moreover, independently of the interpretation of the trait space, it can be used to robustly quantify the dissimilarity between species, which allows us to infer functional diversity from monitoring data.

## Analysis of Baltic Sea Phytoplankton Species

We now turn back to the phytoplankton monitoring dataset (Fig. 1). The data were collected in the Lithuanian coastal area of the Baltic Sea and span a period from May to November for 23 y (1993 to 2015). In total, it contains 730 samples of the biomasses of 516 species measured at different times and stations (Fig. 1*A* shows examples). We analyze the Baltic data using the same procedure that we applied to the simulation results. A projection of the trait space using the most important i-traits is shown in Fig. 1*C*.

**Fig. 3.**
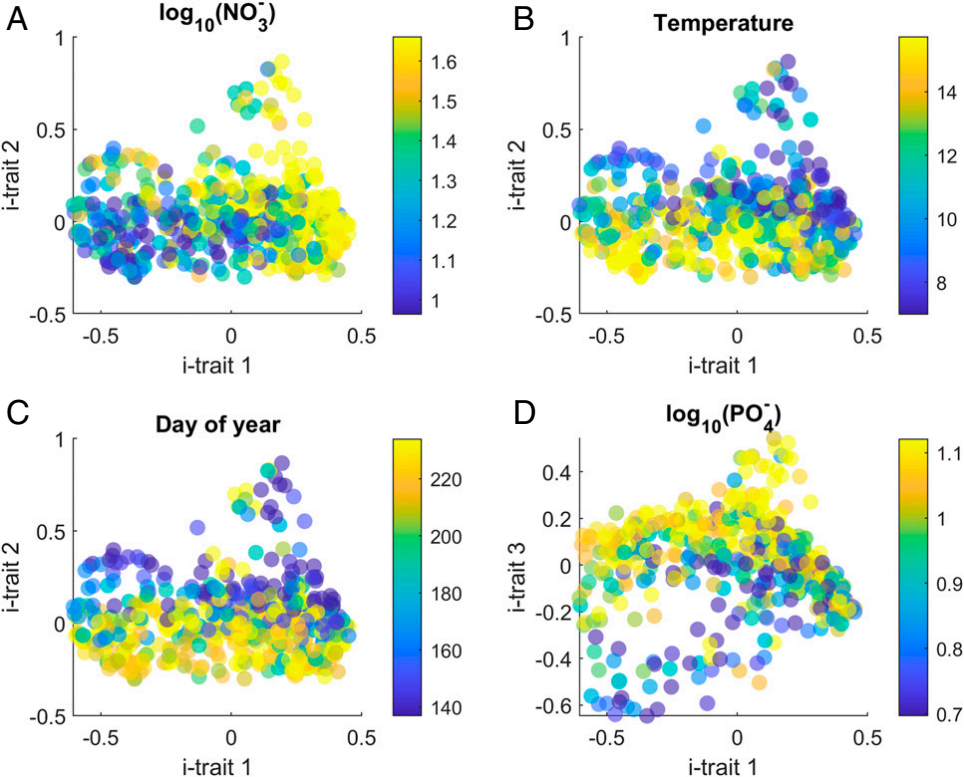
Inferred traits from the monitoring dataset. Shown are species (dots) projected onto the space spanned by the most important i-traits. Color coded are environmental conditions under which the species were observed with high relative abundance (see text). (*A*) The first i-trait aligns well with NO${}_{3}^{-}$ concentration separating species by their nitrogen requirements. The water temperature (*B*) and the day of the year (*C*) align with the second trait, separating the early from the late species. The PO${}_{4}^{-}$ concentration is closely aligned with the third reconstructed trait (*D*).

Diffusion mapping does not provide a biological interpretation of the inferred traits. However, we can uncover such an interpretation for at least some of the traits by analyzing additional data. Here, we discuss in particular four environmental variables that were recorded during sampling (day of year, water temperature, NO${}_{3}^{-}$ concentration, and PO${}_{4}^{-}$). For each of the species, we calculate the mean environmental conditions at which it was observed. This is done by computing a weighted average of each environmental parameter, where the biomass of the species under consideration is used as the statistical weight of the sample. Color coding the species in the reconstructed trait space (Fig. 3) shows that the first i-trait aligns well with NO${}_{3}^{-}$ concentrations (Spearman correlation, $r_{S}=0.55$). We conclude that this trait represents adaptation to different levels of nutrient availability.

We note that the alignment of the i-trait with NO${}_{3}^{-}$ is a purely statistical finding, which does not necessarily imply any causal link. The interpretation as adaptation to different levels of nutrient availability must, therefore, be treated as a working hypothesis (compare to *SI Appendix*, Fig. S12). Similarly, i-trait 2 closely aligns with the temperature ($r_{S}=0.50$) and the day of the year ($r_{S}=0.43$), suggesting that this trait represents the growth strategy, separating early from late species. The third i-trait correlates with the PO${}_{4}^{-}$ concentrations ($r_{S}=0.45$).

The number of i-trait axes equals the dimensionality of the input data (i.e., the number of species). Although each i-trait contains less information than the previous one, projections of the trait space on different trait axes give additional insights into the distribution of species ecotypes (*SI Appendix*).

## Diversity Gains on the Lithuanian Coast

Once the i-trait space has been constructed, it can be used to quantify the functional diversity. We first use the i-traits from the analysis of the whole dataset to compute the diffusion distances between all pairs of species. For each sample, we then use the distances between the species in the i-trait space to compute the Rao index.

**Fig. 4.**
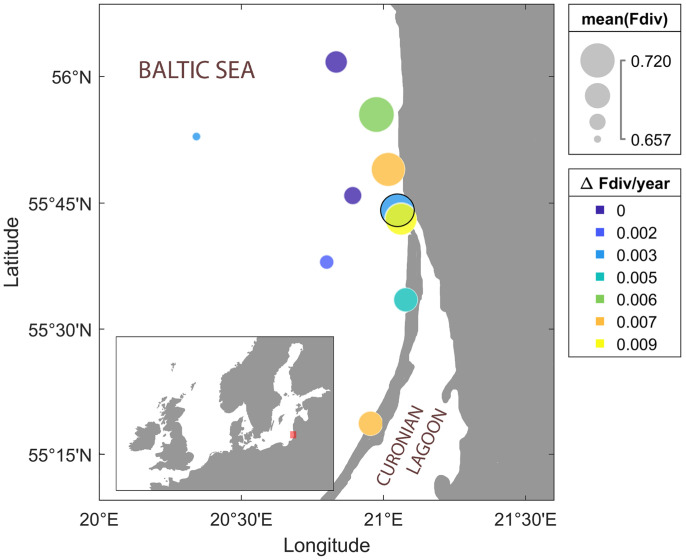
Phytoplankton diversity on the Lithuanian coast. We observe an increase in functional diversity over the measurement period at all of the 10 stations included in the dataset (circles). The station located at the exit of the Curonian Lagoon is marked with a black circle. The fastest increase (warmer colors) is found at some of the coastal stations. The coastal stations are also the most diverse in average (larger diameter).

The estimated day-to-day functional diversities are relatively noisy, likely due to intrinsic fluctuations in the system. However, when considered over the whole period, there is a significant biodiversity gain at all stations (Fig. 4). This gain is most pronounced at the coastal stations, where the functional diversity is also the highest in the later years.

The local increase in functional diversity is consistent with previous findings and predictions. For example, refs. 5, 15, and 30 observe comparable increases in species richness. A key mechanism in this context is that species extinction events triggered by environmental change take longer to manifest than corresponding invasions (15). Hence, environmental disturbances are likely to trigger transient increases in biodiversity on a trajectory that eventually leads to diversity loss when either longer times or larger geographical scales are considered.

In the present case, the difference between coastal and offshore stations provides strong evidence supporting the hypothesis of an invasion-triggered transient increase. The coastal stations are close to the freshwater communities of the Curonian Lagoon. An increased influence of the freshwater species due to changing environmental conditions could easily explain the observed diversity trends. We note that the only coastal station that did not experience a strong increase of functional diversity is located directly at the exit of the Curonian Lagoon (the black circle in Fig. 4) and hence, has always been strongly influenced by the freshwater communities (*SI Appendix*).

The estimated diversity is consistent with expectations based on species composition. The low functional diversity in the spring samples of early 1993 and 2000 (*SI Appendix*, Figs. S14 and S15) coincides with the dominance of dinoflagellates, mainly *Peridiniella catenata*, whose numbers were over 50% of the total phytoplankton abundance, of up to 96% by biomass. During the period of increased functional diversity in spring samples from 1994 to 1999, *P. catenata* was found in small numbers, and the community was dominated by three to five species constituting together of more than 50% of the total abundance. During this time, the number of nondominant species with relative abundance less than 10% also increased.

## Conclusions

In this paper, we proposed a method by which functional trait axes and values can be inferred from monitoring data. This enables a robust estimation of functional diversity within the system based solely on species abundances or biomasses.

We demonstrated the method using simulated data and a phytoplankton monitoring dataset from the Baltic Sea. The analysis of the real-world data identified adaptation for early/late growth, high/low nitrogen levels, and high/low phosphorus levels as the most important functional trait axes. It also showed a local increase in functional diversity that is comparable with previous observations in other systems (5, 15, 30). In the present analysis, the increase is most pronounced at coastal stations and can be linked to increasing influence from a nearby freshwater community. Hence, our results provide additional evidence for the hypothesis that changing environmental parameters may lead to a transient increase in local diversity that might ultimately lead to biodiversity loss on longer and larger scales (5, 6).

We note particularly that the proposed method does not require manual identification of relevant traits. It thus provides an objective and procedurally-grounded definition of functional diversity that is transferable between different systems and sets of species. We expect that this will also be useful in the analysis of other datasets, particularly those that contain a large number of species.

In principle, the proposed method could also be applied to study bacterial diversity. However, in bacteria, the relevant traits are thought to be more closely related to their genetic makeup than in eukaryotes (11). Hence, it is sensible to take the available genomic information into account when constructing diffusion maps of bacteria.

One of the main motivations for the present work was our desire to eventually gain a deeper understanding of the dynamics of complex metacommunities. The trait axes that the proposed method infers can be interpreted as dynamical variables at the community level. Given sufficient data, we may eventually be able to also infer the equations that capture the dynamics directly at this level.

The accuracy of the results should increase with the number of observations, which makes it attractive to combine multiple, and perhaps even all available, datasets for a given group of species. Different monitoring datasets should be relatively easy to fuse at this level because only comparisons within samples from the same dataset need to be made. In the future, a diffusion map based on a large-scale aggregation of many different monitoring datasets could effectively provide a functional diversity standard that can be used to quickly map the functional diversity of samples on a fixed scale.

## Supplementary Material

Supplementary File

## Data Availability

Previously published data from ref. 31 were used for this work.
