# Supplementary material for: Estimation of functional diversity and species traits from ecological monitoring data
Source: Proc Natl Acad Sci U S A. 2022 Oct 18;119(43):e2118156119. doi: 10.1073/pnas.2118156119 (PMC9618138; doi:10.1073/pnas.2118156119)
Supplement: Supplementary File [file pnas.2118156119.sapp.pdf]

Supporting Online Material for Estimation of functional diversity and species traits from ecological monitoring data

Alexey Ryabov, Bernd Blasius, Helmut Hillebrand
Irina Olenina, and Thilo Gross

September 19, 2022

Contents

1 Computational Experiment **1**

2 The why and how of geometric diffusion **4**

3 Diffusion map procedure **8**
3.1 Notation 8
3.2 Similarity matrix 8
3.3 Thresholding 9
3.4 Laplacian 11
3.5 Eigenvectors and eigenvalues 11
3.6 Diffusion distance 11

4 Exploratory Analyses **11**
4.1 Functional diversity 12
4.2 Effect of data availability 12
4.3 Selection of a distance measure 14
4.4 Comparison of diffusion maps and PCA 15
4.5 Mapping dynamical data 16
4.6 Dimensionality of the data space 16

5 Analysis of the Baltic Sea data **20**
5.1 Adaptation to environmental factors 20
5.2 Dynamics of i-traits and functional diversity 25

1 Computational Experiment

In this section we describe the detailed procedure for the simulation of the meta-community model, see [1] for the model analysis. We model spatial competition

of $n = 200$ species for three limiting resources, heterogeneously distributed across a two-dimensional grid comprising of 10×12 diffusively coupled cells. The resource availability in each cell (x, y) is characterized by the equilibrium concentration of the three resources $(S^1_{xy}, S^2_{xy}, S^3_{xy})$ in the absence of consumers.

To obtain samples from environments with varying heterogeneity, we simulated 800 grids with different levels of resource variability. The regional variability of resource r in grid j is constrained by the parameters $S^{jr}_{\min} < S^{jr}_{\max}$, drawn randomly from a uniform distribution in the range from 1 to 39 resource units. The local supply, S^{jr}_{xy}, of resource r in cell (x, y) of grid j is then drawn randomly from a uniform distribution in the range $[S^{jr}_{min}, S^{jr}_{max}]$ in the respective resource grid.

Following the mechanistic theory of resource competition, we use the R^*_{ir} values, i.e., the concentration of resource r at which species i can still persist, as basic species traits [2]. To define the trait of species i, we draw the values R^*_{i1} and R^*_{i2} from a uniform distribution in the range $[0.5, 9.5]$ and then chose the third resource parameter R^*_{i3} such that the sum $\sum_{r=1...3} R^*_{ir} = 19.5$ is constant. We reject parameter combinations for which R^*_{i3} is greater than 9.5. The trait values that are thus selected are uniformly distributed in a triangle in the trait space, as shown in Fig. 2A in the main text.

We model the functional dependence of biomass growth using Monod kinetics of resource uptake and Liebig's law of minimum [3, 4],

$$g_i(\boldsymbol{R}) = g_{\max} \min_{r=1...3} \frac{R_r}{K_{ir} + R_r}, \tag{1}$$

where $g_{\max}$ is the maximum growth rate, R_r is the local concentration of resource r, and K_{ir} is the half-saturation constant of growth of species i limited by resource r. We assume that the maximum growth rate $g_{\max} = 1$ and mortality rate $m = 0.25$ are identical for all species, so that the dimensionality of the species trait space is defined by the R* values only. Based on the condition $g_i(R^*_{ir}) = m$, we can express the half-saturation constants for given values R^*_{ir} as

$$K_{ir} = R^*_{ir} \frac{(g_{\max} - m)}{m}. \tag{2}$$

The system is initialized by assigning random biomass values in $[0, 0.1]$ to each species in each grid cell. These biomass values are small compared to the typical local equilibrium biomass of persisting species.

To describe the evolution of the system, we denote N^i_{xy} as the local biomass of species i, and R^r_{xy} the local concentration of resource r, in cell (x, y). The biomass of species i then evolves according to

$$\frac{\mathrm{d}}{\mathrm{d}t} N^i_{xy} = [g_i(\boldsymbol{R_{xy}}) - m] \cdot N^i_{xy} + \Delta N^i_{xy}, \tag{3}$$

where the first term captures local growth and loss processes and the second term captures dispersal in terms of the discrete Laplace operator

$$\Delta N^i_{xy} = \frac{N^i_{x,y+1} + N^i_{x,y-1} + N^i_{x+1,y} + N^i_{x-1,y} - 4N^i_{xy}}{h^2}, \tag{4}$$

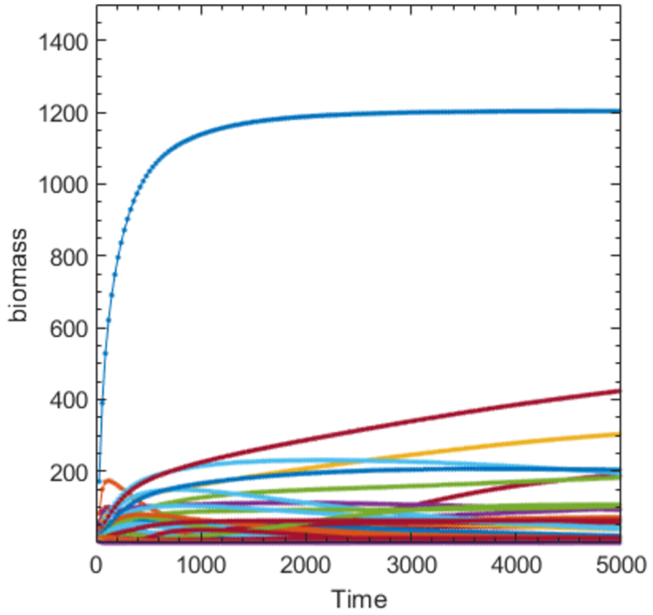

Figure S1: Typical timeseries from a simulation run. Populations approach different stationary levels, however, no competitive exclusion occurs due to the spatial distribution of resources in the metacommunity.

where h is the lattice constant. At the grid boundaries we assume no flux boundary conditions. The dynamics of resource r is given by the difference between local resource inflow and resource consumption (for simplicity, we neglect the diffusion of resources)

$$\frac{\mathrm{d}}{\mathrm{d}t} R_{xy}^{r} = D(S_{xy}^{r} - R_{xy}^{r}) - \sum_{i=1}^{n} c_{ir} N_{xy}^{i}\, g_i(\boldsymbol{R_{xy}}) \tag{5}$$

where c_{ir} is the amount of resource r consumed for the production of one unit of biomass of species i and D is the dilution rate. We assume that c_{ir} is linearly related to R^*, $c_{ir} = 0.05R_{ir}^*$ [1]. This parameterization provides an additional possibility for species coexistence not only due to species diffusion across the grid but also due to local resource partitioning.

We integrate the system numerically for 6000 simulation days. This simulation time is sufficient for species sorting and reducing the effect of initial conditions. If the local biomass of a species drops below 0.01 at the end of the simulation, we regard this species to be locally extinct and set its local biomass to zero (for comparison, the maximum local biomass of species is about 100). However, due to the spatial structure of the system global extinctions did not occur. An example timeserie is shown in Fig. S1.

The local biomass of the 200 competitors at the end of the simulation are then analyzed using the diffusion map procedure described below.

Thus, the raw data includes $10 \cdot 12 \cdot 800 = 96,000$ biomass samples of each of the 200 species. However, diffusion fluxes across cell boundaries lead to biomass correlations in neighboring cells, so that many samples obtained from the same grid are not independent. We estimate the minimal number of independent samples that we use below as the number of simulated grids.

Unless not otherwise specified above, parameter values are taken according to [1]. For numerical integration we use an explicit Runge-Kutta (4,5) algorithm (ode45 solver, MATLAB 2020). The source code for the simulation and data analysis is publicly available at [5].

2 The why and how of geometric diffusion

A central challenge in the analysis of high-dimensional data is to construct faithful metrics for the dissimilarity of distant points in data space. The idea of the diffusion map is to address this challenge by the construction of a network of comparisons that are sufficiently close and can hence be trusted.

Once such a network of trusted links has been identified we can quantify the distance between dissimilar points as the distance on the network. Perhaps the most natural way in which one could do such comparisons is to consider shortest path distances: The distance between any pair of data points is defined as the length of the shortest path on the network of trusted links.

Such a quantification in terms of shortest path distance does indeed work, and provided sufficient data yields very similar results to the diffusion map. However, shortest path distance is very susceptible to noise as it hinges on the presence or absence of specific links. Therefore this notion of distance is typically not used in manifold learning.

To increase the robustness to noise one needs to take the length of alternative (non-shortest) paths between data points into account. For this reason Coifman [6] proposed the notion of diffusion distance, where the distance between points is quantified in terms of properties of random walks on the network. As a process the random walk effectively explores all paths in the network, leading to excellent robustness.

There are some different variants of the diffusion process that each come with their unique advantages and disadvantages. The most common diffusion process studied in physics is continuous time diffusion. In this case walkers move in discrete jumps, but these jumps occur randomly at random times such that the average rate is 1 according to a Poisson process. The probability to find a walker in node i at time t, $x_i(t)$ then changes according to the following differential equation

$$\dot{x}_i = -k_i x_i + \sum_j A_{i,j} x_j, \tag{6}$$

where $A_{i,j}$ is the adjacency matrix of the network and $k_i = \sum_j A_{i,j}$ is the degree of the node. In our case we take this adjacency to be the similarity along trusted links $S_{i,j}$ or zero if there isn't a trusted link between a certain pair of nodes.

The equation above can be written in vector form as

$$\dot{\boldsymbol{x}} = -\mathbf{K}\boldsymbol{x} + \mathbf{A}\boldsymbol{x}, \tag{7}$$

where $\boldsymbol{x} = (x_1, \dots, x_N)^{\mathrm{T}}$, $\mathbf{A} = (A_{ij})$ and $\mathbf{K}$ is the degree matrix defined by

$$K_{i,j} = \begin{cases} k_i & \text{for } i = j \\ 0 & \text{otherwise} \end{cases} . \tag{8}$$

Defining the Laplacian $\mathbf{L} = \mathbf{K} - \mathbf{A}$ allows to write this equation more compactly as

$$\dot{\boldsymbol{x}} = -\mathbf{L}\boldsymbol{x}. \tag{9}$$

The sign of $\mathbf{L}$ was chosen to make the operator $\mathbf{L}$ positive semidefinite. Apart from this unfortunate choice, the equation is closely reminiscent of the diffusion equation in continuous space ($\dot{\boldsymbol{x}} = \triangle \boldsymbol{x}$). Where the Laplace operator ($\triangle$) has been replaced with the Laplacian matrix $\mathbf{L}$. In fact, the Laplacian matrix can be read as the discretization of the second spatial derivative $\triangle$ on a lattice.

Since the diffusion equation is linear we can solve it by eigen-decomposition and hence

$$\boldsymbol{x}(t) = \sum c_n \boldsymbol{v_n} \mathrm{e}^{-\lambda_n t}, \tag{10}$$

where the c_n are the expansion coefficients of an initial state $\boldsymbol{x}(0)$ with respect to the eigenvectors $\boldsymbol{v_n}$ of $\mathbf{L}$, i.e. $\mathbf{L}\boldsymbol{v_n} = \lambda_n \boldsymbol{v_n}$ for all n and $\sum c_n \boldsymbol{v_n} = \boldsymbol{x}(0)$.

Laplacian matrices of connected networks are positive semi-definite matrices that have exactly one eigenvalue $\lambda_1 = 0$, with the corresponding eigenvector $\boldsymbol{v_1} = (1, \dots 1)^{\mathrm{T}}$. All other eigenvalues are positive. Hence, for $t \to \infty$ all nodes will eventually approach the same state. However, during transient dynamics the occupation probabilities x_i of nodes can be different. Specifically, we can write the instantaneous difference between the state of nodes i and j as

$$d_{i,j} = \sum c_n (v_{n,i} - v_{n,j}) \mathrm{e}^{-\lambda_n t} \tag{11}$$

We can now quantify the dissimilarity of two nodes in terms of the integral over their difference $d_{i,j}$ following a small perturbation

$$D_{i,j} = \int_{t=0}^{\infty} \sum c_n (v_{n,i} - v_{n,j}) \mathrm{e}^{-\lambda_n t} = \sum c_n \frac{v_{n,i} - v_{n,j}}{\lambda_n}. \tag{12}$$

We can see here that differences in the direction of the n-th eigenvector are scaled by the factor $1/\lambda_n$. This gives infinite weight to the first eigendirection, but this is not a problem as all $v_{1,i} - v_{1,j} = 0$ for all i, j. We can see that the most important eigendirections are those with positive eigenvalues close to zero. This makes intuitive sense as perturbations that excite the corresponding eigenvectors will decay very slowly and hence have a long impact on the system. Nodes whose coordinates differ strongly in these important vectors will generally be located far apart from each other. The calculation above motivates interpreting $v_{n,i}/\lambda_n$ as the n-th coordinate of node i in a newly defined space, which traces the manifold and is properly scaled for comparisons between nodes.

Despite it's physical grounding the unnormalized Laplacian is rarely used in diffusion maps because it also has some disadvantages. Perhaps most importantly it is a symmetric matrix and hence its eigenvectors are always orthogonal. In a series of tests we found that this limits the eigenvector's ability to align with natural features of the data.

Common implementations of the diffusion map[6] build on the idea of discrete-time diffusion. In this case one considers a system where a walker moves in discrete time intervals by following one of the available links in every time step. This leads to a discrete time evolution equation of the form

$$x_i(t+1) = \sum A_{ij} \frac{x_j(t)}{k_i} \tag{13}$$

where k_j is the weighted degree of node j, i.e. the sum of the similarities along all trusted links connecting to this node. We can write this system in the vector form

$$\boldsymbol{x}(t+1) = \mathbf{L}'\boldsymbol{x}(t) \tag{14}$$

where $L'_{i,j} = A_{i,j}/k_j$. This matrix is closely related to the physical Laplacian $\mathbf{L}$. We can compute it from $\mathbf{L}$ by dividing each row by the diagonal element, subtracting the identity matrix and inverting the sign of the remaining entries. The result is a matrix with eigenvalues in the interval $[-1, 1]$, where the most important eigenvalues are now the ones close to 1. By computing the first passage times between nodes one can rigorously motivate a new coordinate system where the n-th coordinate of node i is now, $v_{n,i}\lambda_n$, where $\boldsymbol{v_n}$ is again the n-th eigenvector [6, 7, 8].

One of the disadvantages of $\mathbf{L}'$ is that time discretization causes some discretization artifacts that can lead to unphysical behavior. For example on a network that consists of a single linked pair, the walker will be on the starting node in all odd steps and on the respectively other node in all even steps, and hence the occupation probability never equilibrates. The artifacts are less pronounced in larger, more generic networks but generally the negative eigenvalues represent such unphysical modes.

Neither L nor L$'$ is intrinsically right or wrong as both can be advantageous or disadvantageous in certain situations. Mathematical work even favors a third Laplacian, the symmetrically normalized matrix

$$L''_{ij} = \begin{cases} 1 & \text{for } i=j \\ -A_{ij}/\sqrt{k_i k_j} & \text{otherwise} \end{cases}, \tag{15}$$

which cannot be motivated by common diffusion processes, but is closely related and has advantageous mathematical properties [9].

In a previous paper[10] we use the row normalized Laplacian

$$L'''_{ij} = \begin{cases} 1 & \text{for } i=j \\ -A_{ij}/k_i & \text{otherwise} \end{cases}. \tag{16}$$

This Laplacian can be seen as a compromise between $\mathbf{L}$ and $\mathbf{L}''$. It can be derived from a physical continuous time diffusion process on a rescaled network.

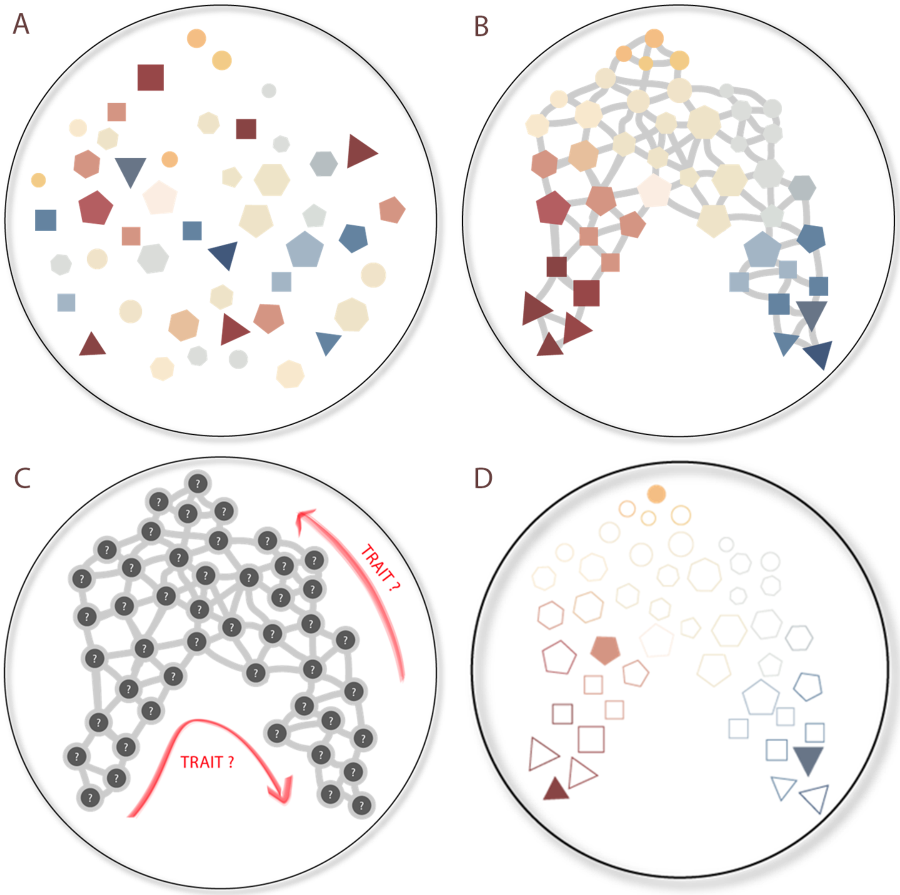

Figure S2: **Illustration of functional diversity mapping.** When dealing with large collections of dissimilar species (symbolized in A) it is useful to identify the network where the links are placed between species that are so similar that they can be faithfully compared (B). Here we construct such a network based solely on the abundance of species in samples. Although no trait information is available, traits can be inferred by identifying the major directions that span the network from the eigenvectors of a diffusion operator (C). Once the entire set of species has been assigned values of the inferred trait, long distance comparisons between species become possible, allowing us to robustly compute the functional diversity (D). In the example (D) a set of four species covers a large part of the variation seen in the whole dataset, hence such a sample would have a high diversity despite the low number of species.

It can thus benefit from the wealth of physical intuition on continuous time diffusion processes. Like $\mathbf{L}$ it has a positive semidefinite spectrum with a single zero eigenvalue (in connected networks). At the same time it's eigenvectors are identical to those of $\mathbf{L}'$.

While working on previous work, including [11, 10] and others, we have at several times compared diffusion maps that were obtained with different variants of the Laplacian. The results where in all cases very similar, but in our experience the matrix $\mathbf{L}'''$ typically yields the best results. We have therefore also used $\mathbf{L}'''$ as the Laplacian for the present paper. Due to it's link to the continuous time diffusion process the appropriate scaling for the new coordinates is the same as for $\mathbf{L}$ such that in new coordinates then nth variable of node i is $v_{n,i}/\lambda_n$.

3 Diffusion map procedure

We now explain the procedure that was used to construct i-traits and to compute diffusion distances between species.

3.1 Notation

We consider m samples containing a subset of a total of n species. The biomass (or abundance) of species i in sample j is denoted by $a_j^{(i)}$. We can then collect all observations of species i in the vector

$$\boldsymbol{a^{(i)}} = \begin{pmatrix} a_1^{(i)} \\ \vdots \\ a_m^{(i)} \end{pmatrix} \tag{17}$$

3.2 Similarity matrix

By comparing different approaches to construct similariteis between species (see below), ee found that the most consistent results are obtained when similarity is determined through the Spearman correlation coefficient. In this case, the similarity of species i and j can be expressed as

$$S_{ij} = r_s(\boldsymbol{a^{(i)}}, \boldsymbol{a^{(j)}}) = \frac{\mathrm{cov}(\boldsymbol{rg_i}, \boldsymbol{rg_j})}{\sigma_i \sigma_j}, \quad i \neq j \tag{18}$$

where $\boldsymbol{rg_i}$ and $\boldsymbol{rg_j}$ are vectors of rank variables for the biomasses of species i and j in different samples, $\mathrm{cov}(\boldsymbol{rg_j}, \boldsymbol{rg_j})$ is the covariance between these variables, and σ_i and σ_j the standard deviations of the rank variables.

As we are not interested in the similarity of a species to itself we set $S_{ii} = 0$. Moreover, as the correlation can be negative we rescale it as $S_{ij} \to (S_{ij} + 1)/2$, which maps the values to the unit interval.

An alternative approach (not used in the results in the maintext) is based on defining species dissimilarity as the distance between vectors of either normalized biomass values $\boldsymbol{b}^{(i)} = \boldsymbol{a}^{(i)}/\sigma_i$ or standardized biomass values, $\boldsymbol{b}^{(i)} = (\boldsymbol{a}^{(i)} - \mu_i)/\sigma_i$, where μ_i is the mean value and σ_i the standard deviation of the biomass of species i across all samples. We compute the dissimilarity d_{ij} between species as the Euclidean distance between these vectors

$$d_{ij} = \sqrt{\sum_k \left(b^{(j)}_{(k)} - b^{(i)}_{(k)}\right)^2} \tag{19}$$

and then define the elements of the similarity matrix, S_{ij}, as the inverse of the dissimilarity, such that

$$S_{ij} = \frac{1}{d_{ij}} \tag{20}$$

for $i \neq j$ and $S_{ij} = 0$ for $i = j$.

3.3 Thresholding

Long distance comparisons are unreliable and constitute a source of noise that can swamp the signal. Hence we want to eliminate all but the the largest entries in the similarity matrix. We do this examining each row of $\mathbf{S}$ and setting all but the 10 largest entries to zero. Thresholding more aggressively has a stronger denoising effect, but risks splitting the network into disconnected components. The value of 10 is often a good middle ground that has been used in several previous publications [10, 12, 11]. We confirmed that for the present dataset the links described by the similarity matrix form a spanning component.

The thresholding procedure can leave us with an asymmetric matrix. For numerical reasons it is generally desirable to maintain the symmetry. We therefore resymmetrize the matrix by the operation

$$S_{ij} \to \max(S_{ij}, S_{ji}) \tag{21}$$

The effect of different choices of thresholds is illustrated in Fig. S3. For the simulated dataset from the paper good results are obtained for a range of thresholds that lies roughly between 5 and 30. At the value of 30 the quality of the reconstruction starts to degrade due to the noise that is introduced by the long-range comparisons, which leads to a loss of shape information, particularly around the tips of the triangle. At 5 the network internally becomes too sparse leading to growing holes within the body of the triangle and eventual fragmentation.

Note also that in the figure the orientation of the triangle changes from panel to panel. This is due to a combination of two effects. First, fundamentally, matrices specify their eigenvectors only up to a factor. Hence if $\boldsymbol{v}$ is an eigenvector with eigenvalues λ (i.e. $\mathbf{L}\boldsymbol{v} = \lambda\boldsymbol{v}$) we can rescale the eigenvector by a factor c and it will still be an eigenvector ($\mathbf{L}(c\boldsymbol{v}) = c\mathbf{L}\boldsymbol{v} = \lambda(c\boldsymbol{v})$). Hence by default a matrix only gives us the direction, but not the length of an eigenvector.

While we impose a normalization condition such that the length of the vector is constrained to 1, this still allows for two different orientations of the vector. In diffusion-mapping we can use information contained in the eigenvalue to recover the best choice for the length of the eigenvector. However, note that c can also be negative, which means the orientation of the eigenvector is still arbitrary and hence can flip between two configurations between trails.

In our test data there is another effect that is due to the peculiarity of the simulated dataset. In the numerical experiments the trait space was chosen as a symmetric triangle, so there are three major axis of near identical length. Hence the first two eigenvectors are of exactly equal importance and their order can change from trial to trial due to numerical effects.

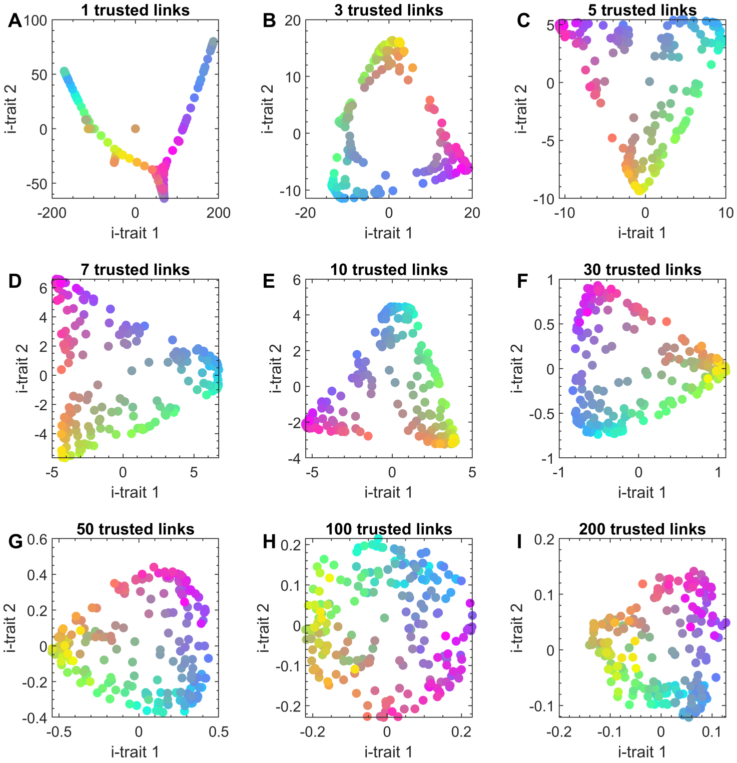

Figure S3: Effects of the number of trusted links in the similarity matrix on the diffusion map. Shown is the i-trait space constructed from similarity matrices with different number of trusted links (see titles). Good results are obtained for a broad range of intermediate threshold values (ca. 5–30). If thresholds are chosen too small the network becomes fragmented. If thresholds are chosen too large the results become noisy. In the different panels the orientation of the reconstructed triangle changes due to a combination of the nature of eigenvectors and peculiarities of the synthetic dataset used in this demonstration (see text).

3.4 Laplacian

From $\mathbf{S}$ we construct the row normalized Laplacian $\mathbf{L}$ defined by

$$L_{ij} = \begin{cases} -S_{ij}/\sum_j S_{ij} & i \neq j \\ 1 & i = j \end{cases} . \tag{22}$$

and solve the eigenvalue problem

$$\mathbf{L}\boldsymbol{v_i} = \lambda_i \boldsymbol{v_i}, \qquad i = 1...n.$$

3.5 Eigenvectors and eigenvalues

All further analysis builds on the eigenvalues and eigenvectors of $\mathbf{L}$. We compute these eigenvalues and eigenvectors numerically using the function eig() from MATLAB 2021, which solves eigenvalue problems using the QR algorithm.

The Laplacian always has at least one eigenvalue at zero. The multiplicity of this eigenvalue is identical to the number of components in the network. In our case, the network has only one component, hence the zero eigenvalue has multiplicity one. We denote this eigenvalue as λ_0. The corresponding eigenvector $\boldsymbol{v}_0$ contains no further information. All other eigenvalues are positive and the corresponding eigenvectors contain trait information.

Since the Laplacian $\mathbf{L}$ is a symmetric matrix, all eigenvalues are real. The eigenvectors corresponding to small eigenvalues are respectively more important, i.e. explain more of the variation along the manifold. Hence the most important eigenvector $\boldsymbol{v_1}$ is the vector corresponding to the smallest non-zero eigenvalue. The eigenvector $\boldsymbol{v_2}$ corresponding to the second smallest non-zero eigenvector corresponds to the second most important trait and so on.

Each eigenvector contains n elements which assign a proxy trait value to each of the n species. As the eigenvalues are inversely related to the importance of the trait we define the value of trait k of species i as $v_{k,i}/\lambda_k$.

3.6 Diffusion distance

Once the trait values have been computed the dissimilarity can be quantified by the distance in trait space. To compare two species we compute the euclidean distance between species in the i-trait space where the species traits are now given by the eigenvector elements corresponding to the species, rescaled by the respective eigenvalue. Hence the distance between two species i, j is

$$d_{ij} = \sqrt{\sum_k \left(\frac{v_{k,i} - v_{k,j}}{\lambda_k} \right)^2} \tag{23}$$

4 Exploratory Analyses

In this section we explain the calculation of functional diversity and present some supporting information on the analysis of the simulated data. Moreover,

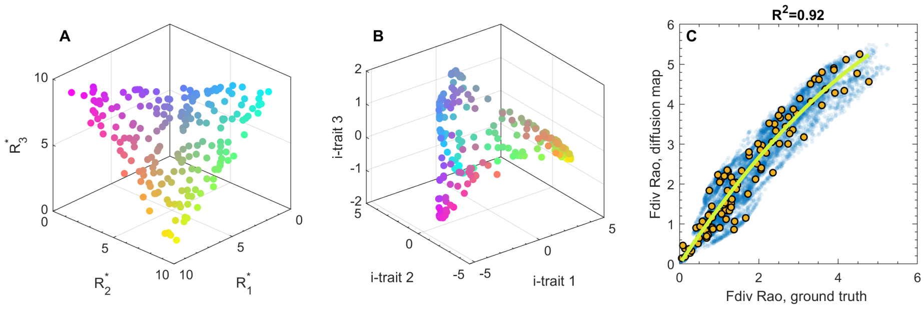

Figure S4: **Embedding using the third infered i-trait.** Same as Fig. 3 in the main text, but with a 3D representation of the species i-traits in B. Note the scale on the third axis. Even if more dimensions are used the inferred manifold remains a two-dimensional object to good approximation. but based on PCA of species similarity matrix calculated as Spearman correlation coefficients.

we present some results from exploratory analysis that lead up to the selection of the Spearman correlation coefficient as our primary similarity measure.

4.1 Functional diversity

There are many ways to assess functional diversity. These methods have various advantages, and the choice of an appropriate index may depend on the specific problem [13]. In this article, we use the Rao's quadratic entropy [14], because this index is sensitive not only to the specific species traits, but also to variation in species abundances. Functional diversity calculated as the Rao index for sample k equals

$$FD_k = \sum_{i=1}^{n-1} \sum_{j=i+1}^{n} d_{ij} p_k^{(i)} p_k^{(j)} \ ,$$

where $p_k^{(i)} = a_k^{(i)} / \sum_j a_k^{(j)}$ is the relative biomass of species i in this sample. This index represents a weighted average functional distance between all pairs of species i and j, where the weighting factor $p^{(i)}p^{(j)}$ is the probability that one of two randomly selected individuals belongs to species i and the other to species j.

4.2 Effect of data availability

In the main text we report the results of trait inference with simulated data from 800 grids. To explore how data availability affects the trait inference we repeat the inference using 100, 200, and 600 grids. For each of these trials we show the i-trait space and the estimated functional diversity (shown for the full data set in the main text), as well as distances between traits measured in terms of R^* values vs. diffusion distances (Fig. S5).

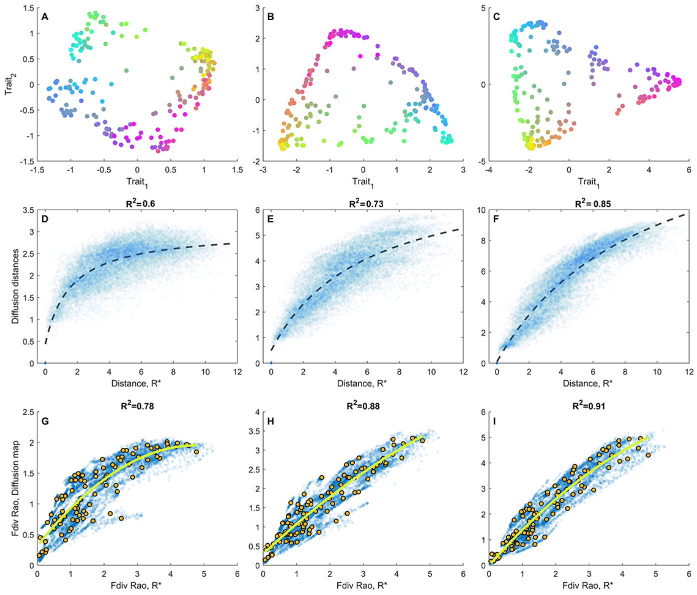

Figure S5: **Effects of data availability.** Shown is the i-trait space (top row), a scatter plot of ground truth (R^*) trait distances vs. i-traits distances (center row), and a scatter plot of estimated functional diversity vs. ground truth functional diversity (bottom row) (*cf.* Fig. 2 from the main text). We compare different results using 100 (left column), 200 (center column), and 600 (right column) simulated grids to produce a diffusion map. The comparison and the indicated R^2 values in the second and third row illustrate how limited data availability degrades the inference result.

Using a small number of 100 grids the inference is very noisy, yielding a square rather than a triangular shaped trait space. The results of the inference improve steadily as we increase the number of grids used. With a number of 600 grids the quality of the inference is comparable to that of the 800-grid result shown in the main text.

The diffusion map based on 100 grids allows us to reliably distinguish only species at small to intermediate functional distances in R^* space, however inferred functional distance fluctuates around a constant level when the R^* functional distance is large (Fig. S5D, G). The correlation between both functional distances and functional diversities measured in R^* space and in the i-trait space increase with the number of samples included, and the relationships approach to monotonically increasing reversible functions (Fig. S5, middle and bottom row). This means that a sufficient input data volume provides a correct mapping for the entire range of functional distances.

4.3 Selection of a distance measure

When the original species traits are known, it is easy to estimate the amount of input data needed to construct a diffusion map with a targeted accuracy. For field data, however, we need an additional method to estimate the uncertainty for a given dataset and the possibility of reducing the uncertainty by increasing the amount of data. To be able to achieve this we suggest to use bootstrapping of samples, a common machine learning method for estimating the rate of uncertainty reduction with the number of training samples. This approach enables us to estimate the uncertainty of diffusion maps and allows the analysis of learning curves.

We denote a similarity matrix constructed by using m samples as $\mathbf{S}(m)$. To calculate this matrix we use bootstrapping, i.e., we select a random set of m out of the n samples with replacement. Thereby, on the basis of n samples we obtain a reduced set of m samples, which will probably include some samples several times, but have statistical properties close to the original set of samples. A bootstrap sample of n out of n samples will include, on average, 63% of the original data, while 37% will remain unused. This approach thus gives an estimate of the uncertainty of the similarity matrix generated from the n samples.

To estimate the uncertainty, we quantify the 'difference' between a similarity matrix based on a subset of m samples and a similarity matrix based the full set of n available samples by the correlation between the elements of these matrices

$$V(m) = \operatorname{cov}(\mathbf{S}(m), \mathbf{S}(n)) \tag{24}$$

Computing $V(m)$ over an increasing size m of the subset yields a "learning curve" (Fig. S6). Constructing such curves using different common notions of distance between species (see Supplement section 2.2) makes it possible to choose the metric that gives the most robust result. In this test the Spearman correlation performed very well, both for simulated and empirical data, yielding a good accuracy with limited data. Based on these results we choose the Spearman correlation as our primary notion of similarity between species.

The value $V(n)$ gives an estimate of the uncertainty in $\mathbf{S}$ when all n samples are used. Extrapolating these curves for $m > n$ provides a rough idea of the number of samples needed to calculate the similarity matrix with the desired accuracy. For the simulated data, when all samples are used, the correlations achieve values from 0.7 to 0.85 depending on the distance metric (Fig. S6A), and the correlation continues to grow monotonically. To obtain a substantial reduction of uncertainty in this case one need at least to double the number of samples. In the field data, compared to the model data, the number of independent variables (species) is larger and the number of observations is smaller. This increases the uncertainty of the similarity matrix, and the correlations obtained for the complete dataset achieve maximal values of $V(m) = 0.6$ (Fig. S6B). Thus, an increase of the monitoring area, frequency, or time-span should be able to significantly improve the accuracy of both the similarity matrix and i-traits.

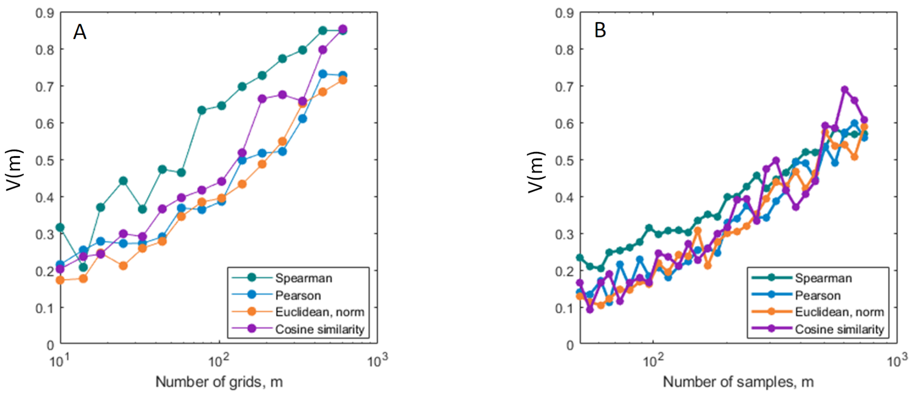

Figure S6: **Learning curve of the similarity matrix for different distance metrics**. In both the simulated data (A) and Baltic Sea samples (B) the Spearman correlation provided the most robust indicator (higher values of $V(m)$) of species similarity over a wide range of values (see text for details).

4.4 Comparison of diffusion maps and PCA

Principal Component Analysis (PCA) is a very common data analysis procedure that identifies directions of large variation in a data cloud. Hence PCA can be used as a simple manifold learning method if the true manifolds in the data are close to linear. However, as we now demonstrate, using PCA for i-trait results in reduced accuracy.

In the analysis of model data significant advantages of the diffusion map over PCA become apparent. We can highlight three main differences. First, the shape of trait distribution in the diffusion map, compared to PCA, is closer to the original distribution shape (cf. Fig. 2A,B in the main text and Fig.S7). Second, the representation of data by diffusion map compared to PCA is much

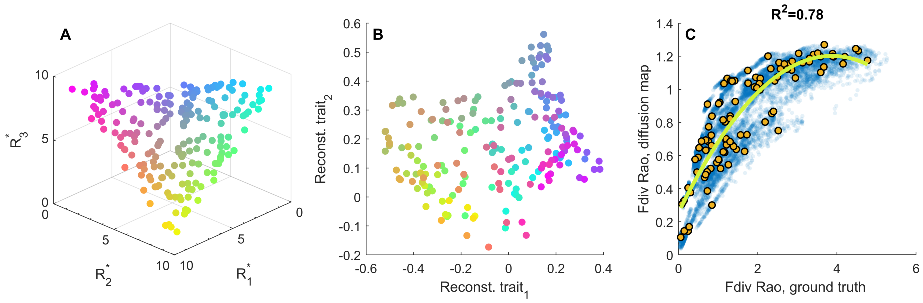

Figure S7: **Poor performance of PCA.** The same as Fig. 2 in the main text but based on PCA of species similarity matrix calculated as Spearman correlation coefficients.

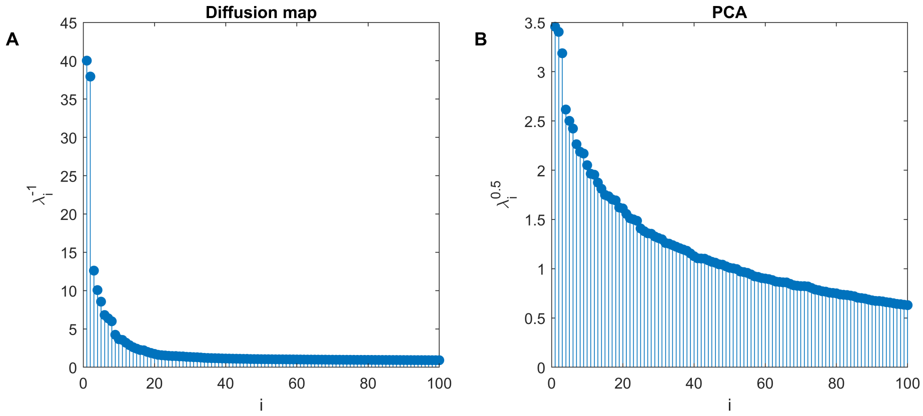

Figure S8: **Comparison of transformed eigenvalues (loadings) calculated for diffusion maps (A) and PCA (B).** The transformed eigenvalue shows the variability in data described by the given axes in the eigenvector space. As show, the representation of data in diffusion map space is much more condense in lower dimension of the eigenvector space, allowing us to use only a few first components to characterize the data.

more condense in lower dimensions (Fig. S8). Finally the fractal dimension of the trait manifold infered by the diffusion map is closer to the dimension of the original manifold than that obtained by PCA (Fig. S10).

4.5 Mapping dynamical data

We also explored whether the stronger ongoing dynamics observed in real data would impinge our ability to construct i-traits. The case can be made that non-stationary dynamics help-rather than hinder the inference as they provide us with more independent information we therefore ran a smaller test using only 100 grid replicas, where resources were reshuffled after 50 time units. Already based on this limited amount of data a comparatively good inference of the trait space was possible (Fig. S9)

4.6 Dimensionality of the data space

To compare the representation of the original trait manifold by a minimal set of i-traits obtained by PCA and diffusion map, it is convenient to use the obtained eigenvalues. For diffusion maps, species traits are defined as $t_{j,i} = v_{j,i}/\lambda_j$, while PCA ordination of species uses the so-called scaling 2 representation where species traits are defined as $t_{j,i} = \sqrt{\lambda_j} v_{j,i}$ [15]. Since the absolute values of the eigenvectors $\boldsymbol{v_j}$ equal one, the comparison of the ranges of species trait variation is reduced to a comparison of the transformed eigenvalues: $1/\lambda_j$ for the diffusion map and $\sqrt{\lambda_j}$ for PCA (Fig. S8). Note that $\sqrt{\lambda_j}$ is termed as loadings in PCA

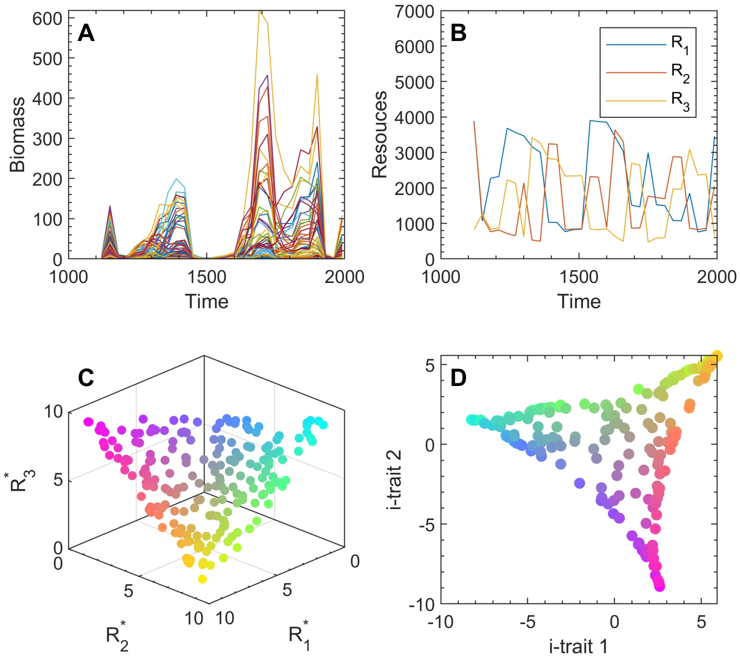

Figure S9: **Trait inference for a dynamical model.** Times series of species (A) and resources (B) remain dynamic in simulations where the resource distribution was updated following the same random process every 50 time units. Grid-averaged species biomass and resource concentrations. (C) Species resource-requirement (R^*). Color indicates the resource ratio preferred by a species. (D) Species i-traits generated by diffusion mapping simulated biomass data. Colors are the same as in C, illustrating that neighborhood relationships are mostly inferred correctly.

analysis.

The initial manifold of species traits is a two-dimensional object (Fig. S7A), and the values of first two inverted eigenvalues obtained by diffusion mapping significantly exceed the subsequent eigenvalues (Fig. S8A), while for PCA this difference is much smaller and even traits defined by eigenvectors with an index greater than 50 carry a significant part of information (Fig. S8B). Thus, diffusion maps compared to PCA are much better at concentrating information about species traits in a lower-dimensional space.

The inferred manifold is a multidimensional object, with the number of dimensions equal to the number of species. However, we expect that the resulting distribution of species traits should keep the original distribution dimensionality and be located on a two-dimensional surface. To estimate the dimensionality of the resulting distribution, we calculated its correlation fractal dimension in the space defined by the first 50 eigenvectors. The fractal dimension of the original

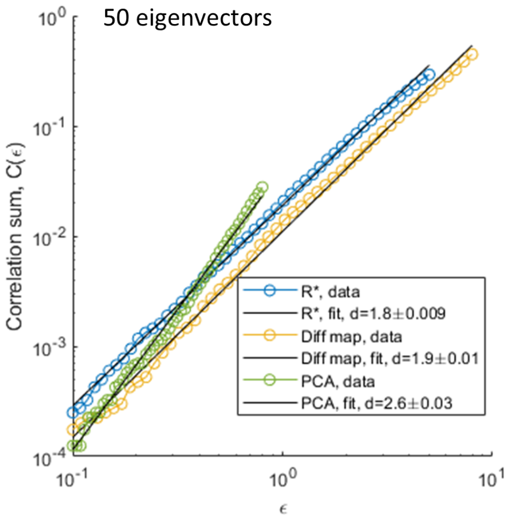

Figure S10: **Estimation of the correlation fractal dimension** d**.** The estimated dimension of the original trait distribution in space R^* is $d = 1.8$, which is close to the actual dimension $d = 2$, since we put all R^* on a plane. The dimensionality of the surface in the i-trait space from the diffusion map is $d = 1.9$. The distribution in PCA space has a larger dimensionality, $d = 2.6$, than the actual distribution, which implies that the data points in PCA space are likely located with random displacement around a two-dimensional surface, effectively creating an object with a fractional dimension greater than 2. The fractal dimension was estimated in the space of the first 50 eigenvectors.

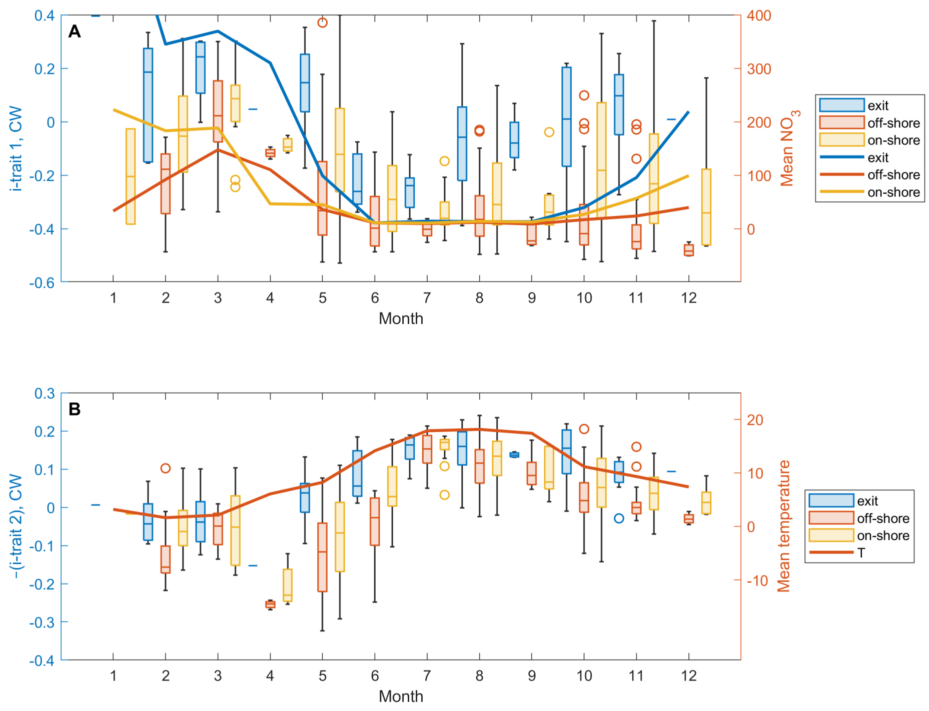

Figure S11: **Seasonal trends in environmental conditions and corresponding community-weighted i-traits.** The box-plot shows the distribution of community weighted mean of i-trait 1 (nitrogen) and i-trait 2 (temperature) for different months and station locations (off shore, on shore, and the Curonian Lagoon exit). Solid lines show the average of NO3 (grouped by locations) and temperature. (A) The variation of i-trait 1 (nitrogen) reaches the highest values in spring and drops to a minimum in summer, corresponding to the period of summer nutrient depletion. Autumn and winter values of i-trait 1 depend on station location, the lowest values being observed for off-shore stations, while species correlating with high nitrogen levels are present at the Curonian Lagoon exit. (B) The variation of the community weighted mean of i-trait 2 is in good agreement with the water temperature, which was almost the same across all locations. As i-trait 2 and temperature are negatively correlated, here we reversed the sign of i-trait 2 to make the comparison clearer. Note that the low i-trait 2 values in April should be considered as an outlier, because the sample in April was taken only in 2000.

distribution, $d = 1.9$, is less than 2, because a random distribution is never perfectly uniform and contains some halls. The diffusion map fractal dimension $d = 1.8$ is close to the original value, but the PCA distribution dimension exceeds it and equals 2.6 (Fig. S10). Implying that the distribution obtained with PCA is closer to a 3D object than a 2D surface.

5 Analysis of the Baltic Sea data

This section contains some additional notes on the Baltic Sea phytoplankton dataset and it's analysis. We use data from at 10 stations the Lithuanian coastal area of Baltic Sea which were regularly visited for 23 years (1993-2015). The data includes 730 samples of biomass of 516 species and environmental data, such as temperature, NO_3, NO_2, PO_4, pH, and salinity.

5.1 Adaptation to environmental factors

Environmental data were measured at different depths for each sample. To obtain a single value for each sample, we interpolated these measurements over a range of depths from 1 to 10 m with resolution of 1 m and calculated the average value in this range.

We estimated species specific environmental condition as the average environmental condition weighted with species biomass

$$\hat{E}^{(r,i)} = \frac{\sum_{j=1}^{n} a_j^{(i)} E_j^{(r)}}{\sum_{j=1}^{n} a_j^{(i)}}$$

where $a_j^{(i)}$ is the biovolume of species i in sample j, $E_j^{(r)}$ is the environmental factor r in this sample, and n is the number of samples. In this way we obtain the species-specific day of year, temperature, nutrients, pH, etc., which are used to colorize the diffusion maps (Fig. 3 in the main text and Fig. S13), and to identify species trait-environment pairs (Fig. S12).

To find the best matching between traits and environmental factors shown in Fig. 3 in the main text, we performed a cross-correlation analysis of the relationships between the environmental factors and the i-traits. We calculated the Spearman correlation between the measured environmental factors and the first ten proxy traits, selected then the trait-environment pair with the highest correlation for each environmental factor, and sorted the results by correlation in descending order. Fig. S12 shows the top nine trait-environment pairs. The first trait correlates with NO_3 ($r_S = 0.55$) and NO_2 ($r_S = 0.49$); the second trait negatively correlates with temperature ($r_S = -0.5$), day of year ($r_S = -0.43$) and positively correlates with salinity ($r_S = 0.32$); the third trait correlates with PO_4 concentration ($r_S = 0.45$) and DIN ($r_S = 0.32$); the fifth trait correlates with NH_4 ($r_S = -0.27$), and the sixth trait with pH ($r_S = -0.34$). We found no strong correlation between the fourth trait and any of the environmental conditions, but this does not mean that this trait is not important, as it may reflect adaptation to factors missed in our data, such as zooplankton abundance, light radiation, or water turbidity.

By projecting the distribution of species traits from the multidimensional diffusion map space onto different species trait axes, we obtain more details about the relationships between environmental factors and proxy traits. This is shown in Fig. S13 which is an extended version of Fig. 3 from the main text. As shown, adaptation to NO_3 correlates only with the first proxy trait,

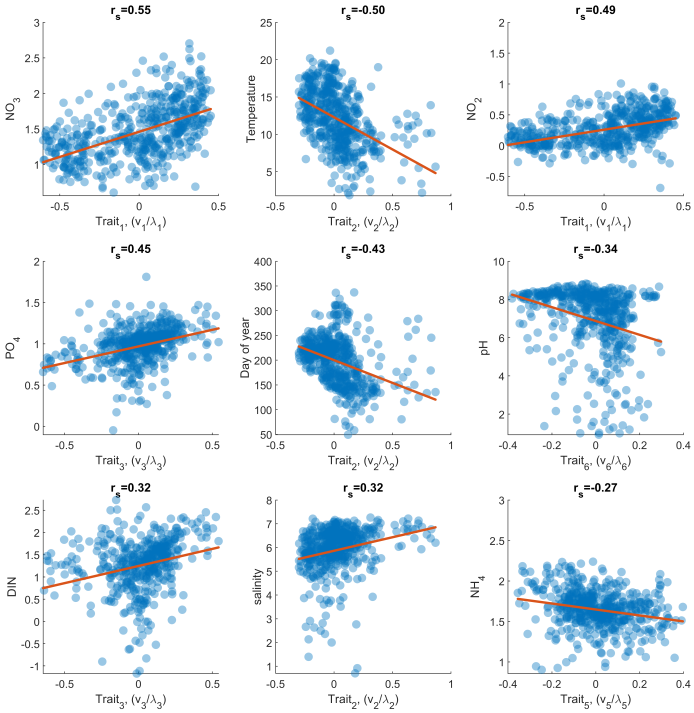

Figure S12: **Correlations between i-traits and species-specific environmental values.** The top nine trait-environment pairs with the highest Spearman correlation coefficient are shown (see text for details).

and adaptation to temperature correlates only with the second trait. At the same time, the day of the year is described by the second, third, and sixth trait (with decreasing correlation). This can be explained by the fact that this parameter strongly positively correlates with temperature, however species dominant in spring and autumn may have the same optimal temperature, but be characterized by different days of the year. Adaptation to PO_4 concentration is also related to several traits, but only the relationship with the third trait is monotonic, while the relationships with the second, fourth, and sixth trait is unimodal.

Fig. S15 shows the dynamics of functional diversity grouped by year (upper row) and additionally by season (lower rows). Functional diversity has remained high on average, with the exception of 1993, 1994, and 2000 (Fig. S15A). Spring samples showed an increase in functional diversity early in the observation period (1993-1999), followed by a decline in functional diversity in 2000, after which functional diversity remained at consistently high levels (Fig. S15B). Extremely large variation between stations, characterized by the size of boxes in Fig. S15, was observed in the spring samples of 1993, 1994, and 1996, in the summer samples of 1994, 1997, and 2001, and also in the fall sample of 2000.

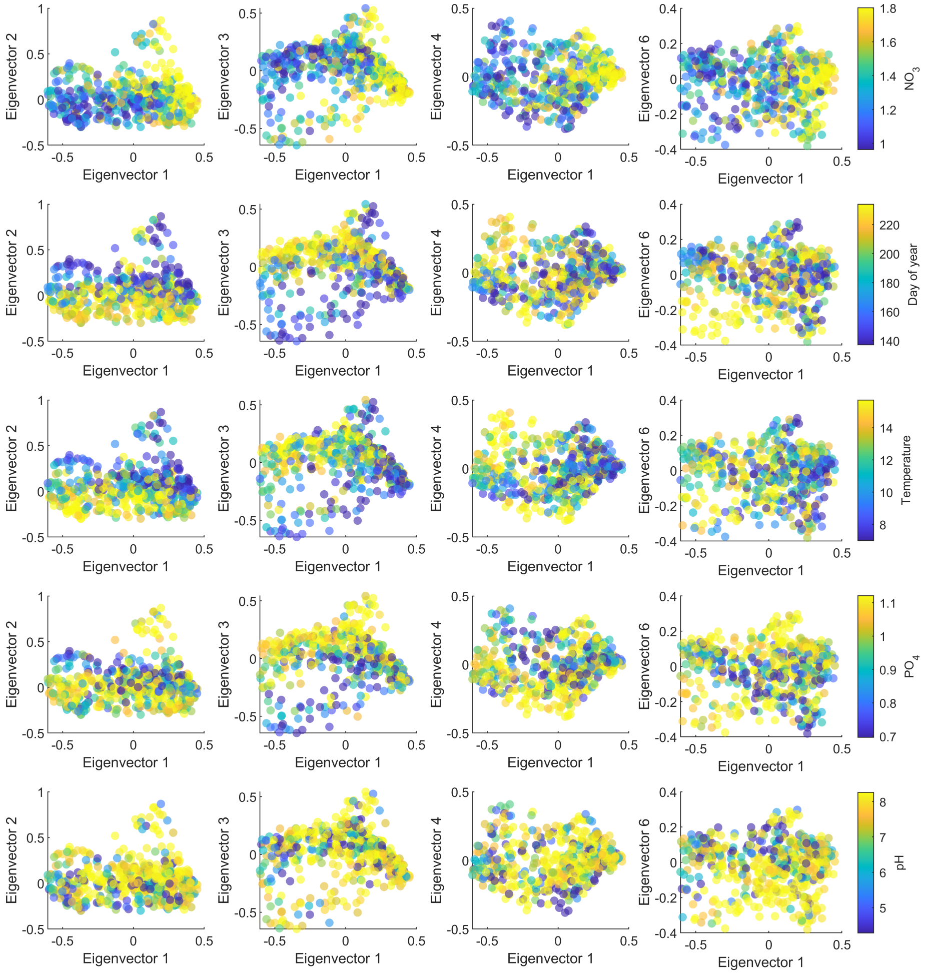

Figure S13: **Projection of diffusion map on different axes.** Color shows the value of the species-specific environmental factor.

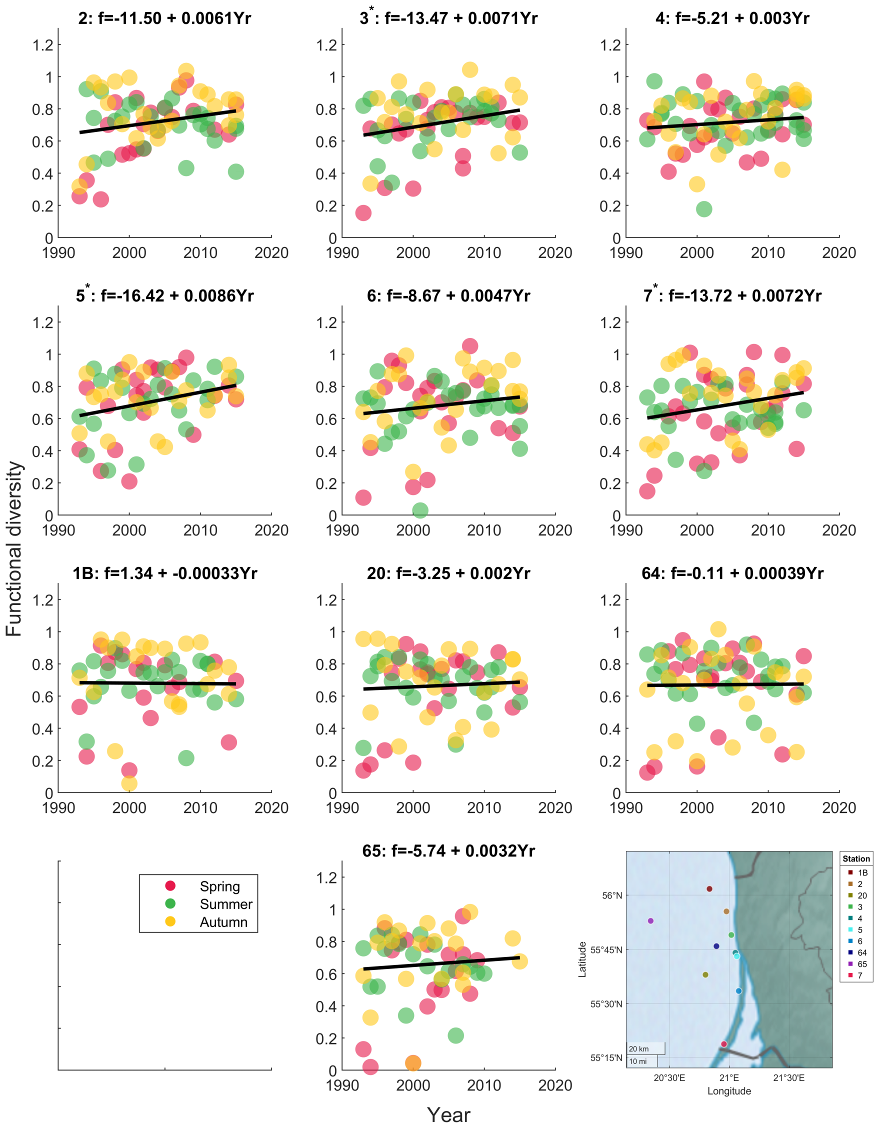

Figure S14: **Temporal trends of functional diversity at different stations.** Functional diversity at coastal stations 2, 3, 5, 6, 7 has increased over the time of observations. This increase was significant ($p < 0.05$) at stations 3, 5, and 7, marked with a star in the plot title. At coastal station 4 it was high during the entire monitoring period. In recent years, samples from all coastal stations exhibit only high functional diversity. By contrast, functional diversity at off-shore stations 1B, 20, 64, and 65 has a weak positive trend and varied over a wide range throughout the observation period.

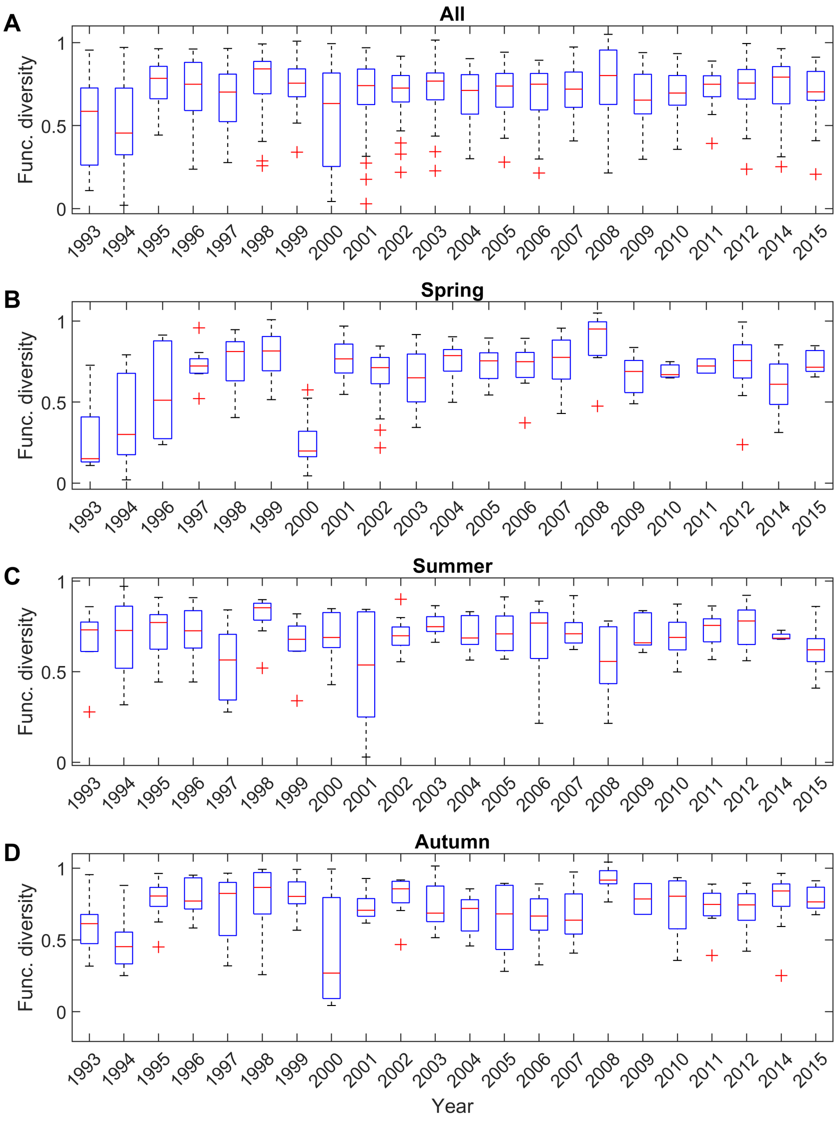

Figure S15: **Temporal trends of functional diversity at different seasons.** Boxplots showing the variation and median values of functional diversity in dependence of the year. The variation in diversity across observations and stations, shown both for the whole year (top row) and separately for each season. The boxplots show the median values (red line), the bottom and top of each box are the 25th and 75th percentiles of the sample, and the whisker length is $\pm 2.7\sigma$ (corresponding to 99.3% for normally distributed data).

5.2 Dynamics of i-traits and functional diversity

For comparison of the dynamics of functional diversity at different stations (Fig. 4, the main text), we carried out a regression analysis of the dependence of functional diversity on the year at different stations (Fig. S14). Since we did not identify a statistically significant seasonal effect on functional diversity, the regression analysis was performed without grouping the data by seasons, but the data were grouped by seasons for clarity when plotting the figure. All coastal stations, with the exception of station 4, show a positive temporal trend in functional diversity, associated with the fact that samples at the beginning of the observations show a wide range of diversity, while samples closer to the end of the observations are characterized mostly by high functional diversity. Coastal station 4, located at the exit of the Curonian Lagoon, is the only coastal station that shows only a small statistically insignificant temporal trend, which is explained by the fact that the functional diversity at this station was at a high level throughout the entire observation period. In contrast to the coastal stations, the samples from off-shore stations (1B, 20, 64, 65) do not show a positive trend in functional diversity and are characterized by a reduced level of functional diversity throughout the entire observation period compared to the coastal stations. The dynamics of some selected i-traits are shown in Fig. S11

References

[1] Hodapp D, Hillebrand H, Blasius B, Ryabov AB (2016) Environmental and trait variability constrain community structure and the biodiversity-productivity relationship. *Ecology* 97(6):1463–1474.

[2] Tilman D (1980) Resources: a graphical-mechanistic approach to competition and predation. *Am. Nat.* 116(3):362–393.

[3] León JA, Tumpson DB (1975) Competition between two species for two complementary or substitutable resources. *Journal of Theoretical Biology* 50(1):185–201.

[4] Huisman J, Weissing FJ (2001) Fundamental unpredictability in multispecies competition. *The American Naturalist* 157(5):488–494.

[5] Ryabov A (2021) Simulation and analysis codes. https://github.com/AlexRyabov/Metacommunity-model; https://github.com/AlexRyabov/Diffusion-map.

[6] Coifman RR, et al. (2005) Geometric diffusions as a tool for harmonic analysis and structure definition of data: Diffusion maps. *Proceedings of the National Academy of Sciences* 102(21):7426–7431.

[7] Coifman RR, Lafon S (2006) Diffusion maps. *Applied and Computational Harmonic Analysis* 21(1):5–30.

[8] Nadler B, Lafon S, Coifman RR, Kevrekidis IG (2006) Diffusion maps, spectral clustering and reaction coordinates of dynamical systems. *Applied and Computational Harmonic Analysis* 21(1):113–127.

[9] Alon N (1986) Eigenvalues and expanders. *Combinatorica* 6(2):83–96.

[10] Barter E, Gross T (2019) Manifold cities: social variables of urban areas in the uk. *Proceedings of the Royal Society A: Mathematical, Physical and Engineering Sciences* 475(2221):20180615.

[11] Fahimipour AK, Gross T (2020) Mapping the bacterial metabolic niche space. *Nature Communications* 11(1):4887.

[12] Ghafourian A, Georgiou O, Barter E, Gross T (2020) Wireless localization with diffusion maps. *Scientific Reports* 10(1):20655.

[13] Legras G, Loiseau N, Gaertner JC, Poggiale JC, Gaertner-Mazouni N (2020) Assessing functional diversity: the influence of the number of the functional traits. *Theoretical Ecology* 13(1):117–126.

[14] Botta-Dukát Z (2005) Rao's quadratic entropy as a measure of functional diversity based on multiple traits. *Journal of Vegetation Science* 16:533–540.

[15] Legendre P, Legendre L (2012) *Numerical Ecology.* (Elsevier).
